# *Physalis peruviana*-Derived 4β-Hydroxywithanolide E, a Novel Antagonist of Wnt Signaling, Inhibits Colorectal Cancer *In Vitro* and *In Vivo*

**DOI:** 10.3390/molecules24061146

**Published:** 2019-03-22

**Authors:** Zhen-Nan Ye, Feng Yuan, Jie-Qing Liu, Xing-Rong Peng, Tao An, Xue Li, Ling-Mei Kong, Ming-Hua Qiu, Yan Li

**Affiliations:** 1 State Key Laboratory of Phytochemistry and Plant Resources in West China, Kunming Institute of Botany, Chinese Academy of Sciences, Kunming 650201, China; zhennan.ye@med.uni-jena.de (Z.-N.Y.); yuanfeng@mail.kib.ac.cn (F.Y.); liujieqing@hqu.edu.cn (J.-Q.L.); pengxingrong@mail.kib.ac.cn (X.-R.P.); antao@mail.kib.ac.cn (T.A.); lixue@mail.kib.ac.cn (X.L.); 2 University of the Chinese Academy of Sciences, Beijing 100049, China; 3 Present Address: Department of Biochemistry II, Jena University Hospital, 07743 Jena, Germany

**Keywords:** 4βHWE, Wnt signaling pathway, β-catenin, colorectal cancer

## Abstract

Deregulation of the Wnt signaling pathway leads to colorectal cancer progression. Natural dietary compounds serve as promising candidates for development as chemopreventive agents by suppressing the Wnt/β-catenin signaling pathway. *Physalis peruviana*-derived 4βHWE showed a significant inhibitory activity with a calculated IC_50_ of 0.09 μΜ in a screening of novel inhibitors of Wnt signaling with the dual-luciferase reporter assay. This study investigated the anti-tumor effect of 4βHWE and the potential Wnt signaling inhibitory mechanism. Both the western blot analysis and immunofluorescence assay showed that 4βHWE promoted the phosphorylation and degradation of β-catenin and the subsequent inhibition of its nuclear translocation to attenuate the endogenous Wnt target gene expression in colorectal cancer (CRC) cells. The cell viability assay indicated that 4βHWE preferentially inhibited the proliferation of CRC compared with CCD-841-CoN, a normal human colonic epithelial cell line. 4βHWE-mediated G0/G1 cell cycle arrest and apoptosis induction contributed to the suppression of the proliferation of CRC in the cell cycle and Annexin V-FITC/Propidium Iodide apoptosis analysis. Moreover, *in vivo*, 4βHWE dramatically inhibited tumor growth in HCT116 xenografts by attenuating the Wnt/β-catenin signaling pathway. In conclusion, our study suggested that 4βHWE could be of potential use in anti-tumor agent development as a novel Wnt signaling inhibitor.

## 1. Introduction

The highly evolutionarily conserved Wnt/β-catenin signaling pathway is integral in embryonic development, the regeneration of tissues in adult organisms, stem cell differentiation, and tissue homeostasis, as well as many other biological processes [1]. Wnts are secreted glycoproteins that trigger a great amount of cellular responses upon activation. The core of the Wnt signaling pathway is the degradation of the transcription co-factor β-catenin mediated by the destruction complex, consisting of Axin, casein kinase 1 (CK1), glycogen synthase kinase-3β (GSK-3β), and adenomatous polyposis coli (APC). In the absence of Wnt ligands, the destruction complex mediates the phosphorylation of β-catenin sequentially by CK1 and GSK-3β and targets β-catenin for ubiquitination and continuous proteasome-mediated degradation to keep the low β-catenin level in the cytoplasm. Once bound by Wnt, the degradation pathway is inhibited, and the phosphorylation and degradation of β-catenin is suppressed. The accumulation of β-catenin leads to its nuclear translocation and complex formation with the transcription factors, T-cell factor (TCF)/Lymphoid enhancer-binding factor (LEF), in the nucleus to regulate the expression of proteins required for cell proliferation and survival, such as cyclin D1, c-Myc, and the anti-apoptotic protein survivin [2,3,4]. 

The Wnt/β-catenin signaling pathway is critically involved in the initiation and progression of various cancers, especially colorectal cancers (CRCs), due to the mutation of APC, Axin, and the *CTNNB1* gene encoding β-catenin [5,6]. Subsequent overexpression of the Wnt downstream target genes that function in cell growth (cyclin D1 and c-Myc) and epithelial–mesenchymal transition (EMT) (E-cadherin and matrix metalloproteases) further promote the malignancy of tumor growth and metastasis [7]. Wnt/β-catenin signaling has become a novel and promising therapeutic target in cancer treatment, and the manipulation of the Wnt/β-catenin signaling pathway has been suggested as a valid treatment option for CRC. 

Recently, naturally derived compounds represent an attractive class of compounds for chemopreventive agent development. Several natural dietary compounds have been suggested as promising alternative options for their ability to inhibit cancers by suppressing the Wnt/β-catenin signaling pathway, such as Genistein, Epigallocatechin gallate, Curcumin, and Resveratrol [8]. *Physalis peruviana* (golden berry) is an edible plant used as a diuretic and treated for malaria, asthma, hepatitis, dermatitis, and rheumatism in folk medicine [9,10]. *Physalis peruviana*-derived 4β-Hydroxywithanolide E (4βHWE) is a natural withanolide with a 17α-oriented side chain [11,12], exhibiting anti-inflammatory effects in diabetic mouse adipose tissue [13] by attenuating NF-κB signaling and obvious anti-tumor activity against various cancer cells [14,15,16,17,18,19]. Further mechanism studies suggested that 4βHWE-induced cytostatic activity was associated with DNA damage [17,18,19], the generation of reactive oxygen species (ROS) [18], apoptosis [17,18], and cell cycle arrest [17]. Despite the promising anti-tumor actions of 4βHWE, the detailed mechanisms of these biological effects remain generally unknown in colorectal cancers. 

In this study, we explored the effect of 4βHWE on Wnt signaling and the growth of CRC cells. Our results suggested that 4βHWE might be a potential inhibitor of Wnt signaling and could be developed as a chemopreventive agent for CRCs.

## 2. Results

### 2.1. 4βHWE is A Potent Inhibitor of the Wnt/β-Catenin Pathway

**Figure 1:**
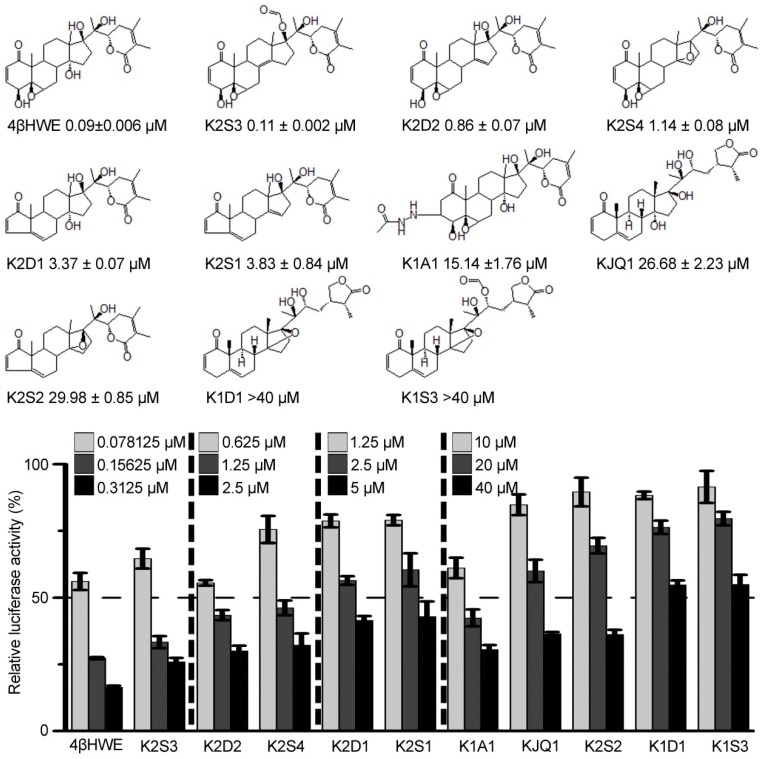
Identification and structure–activity relationship of 4βHWE as a novel Wnt signaling antagonist. HEK293 cells stably transfected with SuperTopflash luciferase (ST-Luc), Wnt3a, and Renilla cells were incubated with 4βHWE or its analogs for 24 h. The inhibitory activity of the compounds was analyzed. The values represent the mean ± SD (*n* = 3). The half ST-Luc inhibition (IC_50_) values were calculated, and the data represent the mean ± SD from three independent experiments.

To identify novel antagonists of the Wnt/β-catenin pathway, a cell-based screening strategy of 4000 compounds from the natural product and natural product-like library (information available from the Big Data Center for Natural Products, State Key Laboratory of Phytochemistry and Plant Resources in West China, Kunming Institute of Botany) was conducted using the dual-luciferase reporter assay in HEK293 cells stably transfected with SuperTopflash luciferase (ST-Luc), Wnt3a, and Renilla [20]. The screening yielded several hits, among which 4βHWE exhibited a strong inhibitory activity in the initial screening and re-screening and was subjected to further study, with IC_50_ of 0.09 ± 0.006 μΜ (Figure 1). The structure–activity relationship analysis suggests that the basic structural requirement for the Wnt signaling inhibitory activity of 4βHWE is the *α*,*β*-unsaturated cyclohexenone at C-1, C-2, and C-3 and a hydroxyl group at C-4 in A ring, as well as 5,6-epoxyl group in B ring. Thus, the hydrogenation of the conjugated fraction and/or A ring shrinkage, as in K1A1, K2D1, K2S1, and K2S2, lead to a decrease of their Wnt inhibitory activities. In addition, the reduction of 5,6-epoxyl results in lower inhibition activities, as in K2D1, K2S1, KJQ1, K2S2, K1D1, and K1S3. Additionally, three free hydroxyl groups at C-14, C-17, and C-20 also play an important role in inhibiting the growth of tumor cells. Compared to K2D3, K2D2, and K2S4, any substitution, reduction, and cyclization could affect their inhibitory activities. Moreover, when an *α*,*β*-unsaturated *δ*-lactone in the side chain is converted to the *γ*-lactone, as in KJQ1, K1D1, and K1S3, the Wnt signaling inhibitory activities of these compounds decrease significantly. 

### 2.2. 4βHWE Interferes with the Wnt Signaling in Colorectal Cancer Cells

**Figure 2:**
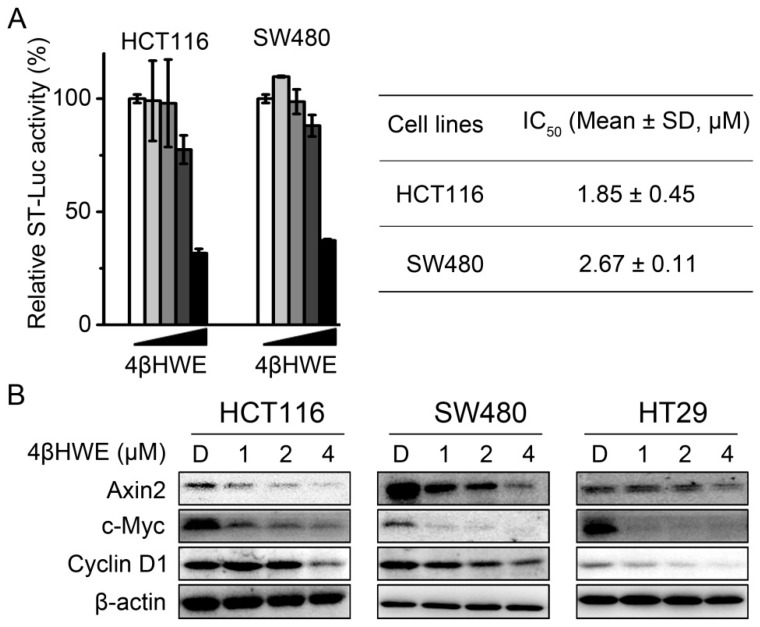
4βHWE suppresses the endogenous Wnt signaling pathway in colorectal cancer (CRC). (**A**) HCT116 and SW480 cells were treated with 0.0064, 0.032, 0.16, 0.8 and 4 μΜ of 4βHWE for 24 h, and the Wnt signaling inhibitory activity of 4βHWE was measured with the dual-luciferase reporter assay. The values represent the mean ± SD (*n* = 3). (**B**) Western blot analysis of lysates from HCT116, SW480, and HT29 cells treated with DMSO (D), 1, 2 and 4 μΜ 4βHWE for 24 h.

Deregulation of the Wnt signaling pathway contributed to tumorigenesis of human CRC; the inhibitory activity of 4βHWE on the Wnt signaling in CRC was studied with the dual-luciferase reporter assay. As shown in Figure 2A, the Wnt signaling activity was strikingly attenuated in HCT116 and SW480 cells, and the calculated IC_50_ values were 1.85 ± 0.45 μΜ and 2.67 ± 0.11 μM in HCT116 and SW480 cells, respectively. Further western blotting results showed that the endogenous Wnt responder gene expression of cyclin D1, c-Myc, and Axin2 was suppressed in HT29, HCT116, and SW480 colorectal cancer cells treated with 4βHWE (Figure 2B). The above results further confirmed 4βHWE as an inhibitor of Wnt signaling and deserving of further investigation.

### 2.3. 4βHWE Inhibits the Stability and Nuclear Translocation of β-Catenin in CRC

**Figure 3:**
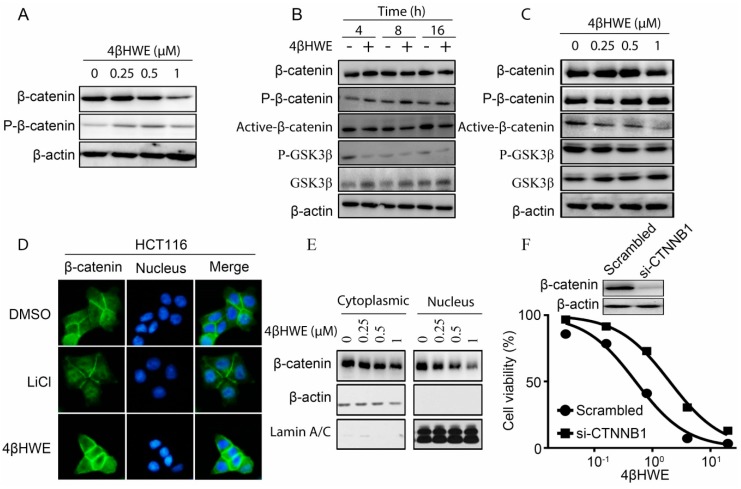
4βHWE disrupts the stability and nuclear translocation of β-catenin in colorectal cancer cells. (**A**) HEK293 cells stably transfected with Wnt3a were treated with 4βHWE for 24 h; β-catenin and p-β-catenin (Ser33, Ser37, and Thr41) were detected with western blot analysis. (**B**) HCT116 cells were treated with 1 µM of 4βHWE for 4 h, 8 h, and 16 h or indicated concentrations of 4βHWE for 16 h respectively (**C**), and western blot analysis of β-catenin, p-β-catenin (Ser33, Ser37, and Thr41), active-β-catenin, p-GSK3β (Ser9), and total GSK3β was performed. (**D**) HCT116 cells were incubated with or without 4βHWE (1μΜ). The β-catenin distribution was detected with the immunofluorescence assay. The green and blue fluorescence represent β-catenin and the nucleus, respectively. (**E**) HCT116 cells were incubated with 0, 0.25, 0.5, and 1 μΜ of 4βHWE for 16 h. The levels of cytoplasmic fraction and nucleic fraction β-catenin in the 4βHWE-treated HCT116 cell lysates were analyzed by western blot. (**F**) The SW480 cells were transfected with *CTNNB1* small interfering RNA for 36 h, and the proliferation inhibitory activity of 4βHWE was measured with the cell viability assay. The knock-down efficiency was analyzed by western blot.

The activity of the Wnt/β-catenin signaling pathway is largely dependent on the level of β-catenin, the core molecule in the pathway [21], the stability of which is regulated by the phosphorylation and degradation of β-catenin by the proteasome [22]. Thus, to investigate the underlying mechanism that 4βHWE inhibits in Wnt signaling, we detected the effect of 4βHWE on the expression and phosphorylation levels of β-catenin. HEK293 cells stably transfected with Wnt3a were treated with 4βHWE for 24 h, and the cell lysates were subjected to western blot analysis. A significant increase in the phosphorylation of β-catenin on Ser33/37/Thr41 residues and the reduction of the total level of β-catenin were observed (Figure 3A). Consistently, HCT116 cells incubated with 4βHWE with incremental time and concentration led to an increase of phospho-β-catenin and a decrease in both the active nonphosphorylated form and the total β-catenin (Figure 3B,C) in western blot analysis. As the destructive complex consisted of GSK3β/Axin/APC/CK1, the cytoplasmic β-catenin level was maintained at a low level [1]. GSK3β functions as a negative regulator of the Wnt signaling pathway through controlling the phosphorylation and degradation of β-catenin [23]. The decreased expressions of the inactive status GSK3β (Ser9) and the accumulation of the total level of GSK3β protein in HCT116 cells were observed in cells treated with 4βHWE (Figure 3B,C). 

Accumulation of phosphorylated β-catenin led to the subsequent nuclear translocation inhibition of β-catenin. The immunofluorescence assay was carried out to analyze the distribution of β-catenin. As shown in Figure 3D, as an inhibitor of GSK-3β [24], LiCl induced the nuclear translocation of β-catenin, while the endogenous nuclear translocation of β-catenin in HCT116 cells was suppressed by 4βHWE. Consistently, the level of β-catenin in the cytoplasmic and the nucleic fraction of HCT116 cells was attenuated when treated with 4βHWE in the western blotting assay (Figure 3E). 

To further investigate the role of the β-catenin/TCF transcription pathway in 4βHWE-mediated anti-tumor activity, the proliferation inhibitory activity of 4βHWE in SW480 cells, with β-catenin knocked down using small interfering RNA, was detected. As shown in Figure 3F, the siRNA-mediated knock-down of β-catenin reduced the sensitivity of cells to 4βHWE, further confirming that β-catenin/TCF is required for the anti-tumor activity of 4βHWE, in part at least.

### 2.4. 4βHWE Selectively Suppresses the Proliferation of CRC

**Figure 4:**
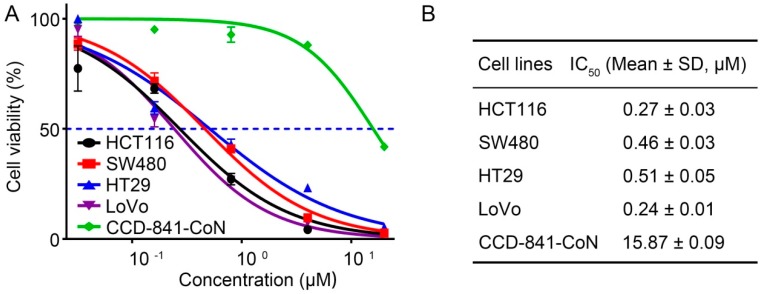
4βHWE selectively suppresses the proliferation of CRC. (**A**) The proliferation inhibition of 4βHWE on CRCs, including four colorectal cancer cell lines (HCT116, SW480, HT29, and LoVo) and CCD-CoN-841, a normal human colonic epithelial cell line, was determined by MTS assay. Cells were treated with 4βHWE for up to 48 h. (**B**) The median inhibition concentration (IC_50_, 48 h) values of 4βHWE for various cell lines were calculated.

The proliferation inhibition effects of 4βHWE on CRC were evaluated with MTS assays in four cancer cells, including HCT116, SW480, HT29, and LoVo, as well as CCD-841-CoN (a normal human colonic epithelial cell line). As shown in Figure 4A, 4βHWE led to marked growth inhibition of HCT116, SW480, HT29, and LoVo, and the IC_50_ values were 0.43 ± 0.03 μΜ, 0.52 ± 0.03 μΜ, 0.49 ± 0.05 μΜ, and 0.29 ± 0.01 μΜ, respectively (Figure 4B). Notably, 4βHWE exhibited lower toxicity to normal colonic epithelial cells (CCD-841-CoN) (the green line in Figure 4A) in comparison with CRC cells, with a calculated IC_50_ value of 1.24 ± 0.09 μΜ, suggesting that 4βHWE selectively exhibited growth inhibition on CRC.

### 2.5. 4βHWE causes G0/G1 Cell Cycle Arrest and Induces Apoptosis in CRC

**Figure 5:**
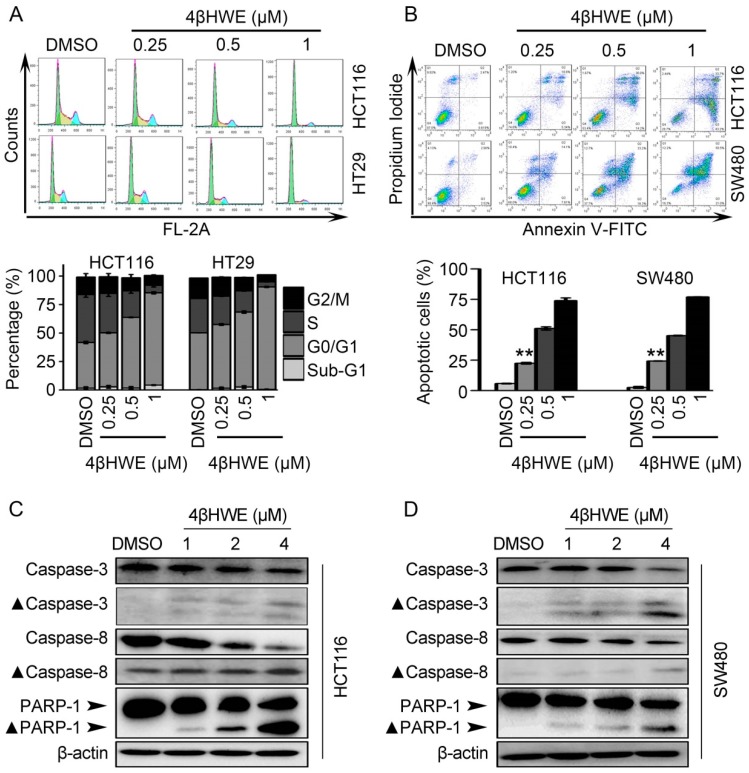
4βHWE induced G0/G1 cycle arrest and apoptosis in CRC. (**A**) HCT116 and HT29 cells were treated with indicated concentrations of 4βHWE for 24 h respectively, and the distribution of the cell cycle was documented. (**B**) Cells were treated with 4βHWE for 48 h, stained with Annexin V-FITC and PI, and analyzed by flow cytometry. *P*-values were derived from the Student’s t-test (* *p* < 0.05, ** *p* < 0.01). HCT116 (**C**) and SW480 cells (**D**) were treated with DMSO, 1, 2, and 4 μΜ of 4βHWE for 24 h, and the lysates were subjected to western blot analysis. The level of apoptosis-related proteins was detected.

To explore the mechanism of 4βHWE suppressing the proliferation of CRC, cell cycle distribution and cell apoptosis induced by 4βHWE were investigated with the propidium iodide (PI) and Annexin V-FITC/PI double staining respectively. As shown in Figure 5A, 4βHWE caused G0/G1 cycle arrest in HCT116 and HT29 cells. The percentages of G0/G1 cells in HCT116 and HT29 cells were 40.04% and 49.93% in cells treated with DMSO, and the numbers run up to 81.04% and 89.99% with 1 μM 4βHWE treated for 24 h, respectively. In the apoptosis assay, as shown in Figure 5B, the percentage of apoptotic cells drastically went up in a dose-dependent manner in 4βHWE-treated HCT116 and SW480 cells. The proportions of apoptotic cells in HCT116 and SW480 cells were 5.77% and 2.56%, respectively, which dramatically increased to 73.93% and 77.00% in 1 μM 4βHWE when incubated for 48 h. We further confirmed that proteolytic cleavages of caspase 3, caspase 8, and PARP-1 were involved in 4βHWE-induced apoptosis in HCT116 and SW480 cells with the western blotting assay (Figure 5C,D).

### 2.6. 4βHWE Suppresses In Vivo Tumor Growth Through Downregulation of Wnt Signaling

The tumor inhibitory activity of 4βHWE was further evaluated in vivo using a BALB/c-nude mouse HCT116 cell xenograft model. Briefly, 4 × 10^6^ HCT116 cells were implanted subcutaneously to the right flank of the mice to develop murine xenograft models. Five days later, the HCT116 xenografts borne by the BALB/c mice were randomly assigned into a control and an experimental group. The mice were treated daily with normal saline as controls or with 5 mg/kg and 10 mg/kg of 4βHWE by intraperitoneal injection.

**Figure 6:**
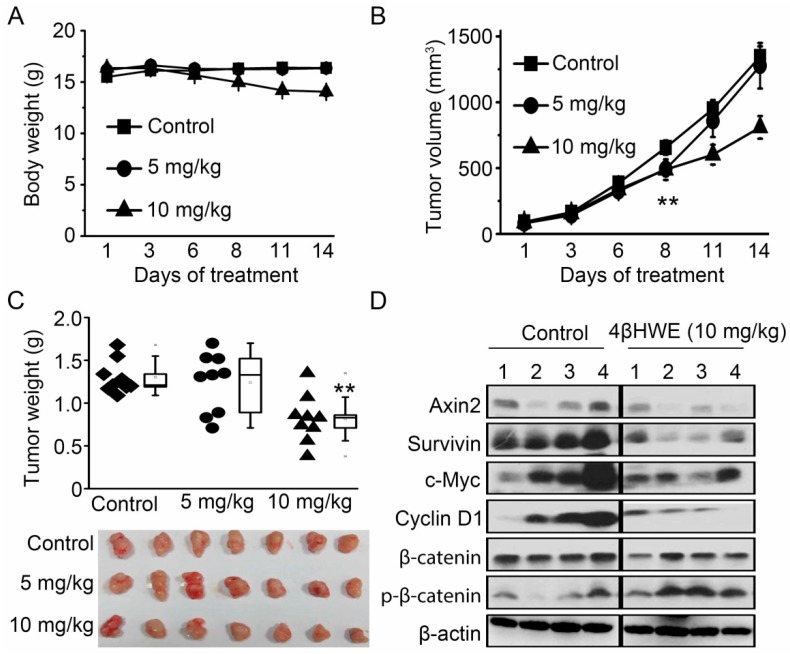
4βHWE inhibits tumor growth of in vivo HCT116 xenograft through Wnt signaling. (**A**) 4 × 10^6^ HCT116 cells were injected into BALB/c-nude mice, and normal saline was given as a control or 4βHWE was injected at a dose of 5 mg/kg and 10 mg/kg/day for 14 days. The body weight was measured as the treatment started. (**B**) The individual HCT116 xenograft tumor volume was monitored. (**C**) The weight of each tumor was measured on the 14th day, and the average tumor weight was calculated. The representative external appearance of the tumors are shown upon normal saline or 4βHWE treatments. (**D**) The effect of 4βHWE on the expression of Wnt-related proteins in tumor tissues was determined by western blot analysis.

As shown in Figure 6A, a daily intraperitoneal administration of 4βHWE at the dose of 5 mg/kg hardly changed the tumor volume due to the quick tumor growth, while 10 mg/kg of 4βHWE dominantly attenuated the growth of the human colon cancer HCT116 xenograft with slight body weight loss, as compared to the control group (Figure 6B). We then sacrificed the animals and excised the tumors on the 14th day for weight measurement and subsequent western blot analysis. The tumors of mice treated with 10 mg/kg 4βHWE were approximately 809 mm^3^, while the tumors in the control group grew to approximately 1347 mm^3^, and a dominant reduction in tumor weight was observed (Figure 6C). Further western blot analysis results showed that the expressions of the Wnt target genes Axin2, c-Myc, survivin, and cyclin D1 were significantly downregulated in the tumor tissues treated with 4βHWE (Figure 6D), with a slight decrease of β-catenin and an increase of Ser33/Ser37/Thr41 phosphorylation of β-catenin. Collectively, these data indicated that 4βHWE inhibited in vivo Wnt signaling in HCT116 xenograft tumors, consistent with our in vitro data.

## 3. Discussion

There is convincing evidence that deregulation of Wnt/β-catenin signaling contributes to the progression of colorectal cancers [25]. In this study, we found that 4βHWE was a potential dietary chemopreventive agent against HEK293 cells with overexpressed Wnt3a, and α,β-unsaturated ketone of A ring and β-OH at γ position were demonstrated to be the main functional groups in a further structure–activity relationship analysis. Based on our results, 4βHWE treatment suppressed the β-catenin/TCF transcriptional activity and the Wnt downstream target gene expression of cyclin D1, c-Myc, and Axin 2 in CRC. Our mechanism study elucidated that 4βHWE increased β-catenin phosphorylation and disrupted the stability of β-catenin and its subsequent nuclear translocation. Further studies on the β-catenin destruction complex were performed to investigate the mechanism of 4βHWE. 4βHWE effectively inhibited the phosphorylation of GSK3β (Ser9), leading to an accumulation and activation of GSK3β and subsequent degradation of β-catenin and its nuclear translocation inhibition. The involvement and association of GSK3β in the regulation of Wnt signaling makes it an attractive therapeutic target in the development of Wnt antagonists [26], such as β-carboline alkaloid compound 9-hydroxycanthin-6-one, inhibiting Wnt signaling by activating GSK3β [27].

Suppression of the Wnt signaling pathway can contribute to the inhibition of cell growth of colorectal cancer cells [28,29]. In conclusion, 4βHWE selectively exhibited a growth inhibitory effect on CRC cells compared with CCD-841-CoN, a normal human colonic epithelial cell line. 4βHWE-induced G0/G1 cell cycle arrest and caspases-mediated apoptosis led to in vitro inhibition of the CRC cell proliferation. Of note, 4βHWE treatment suppressed the expression of the c-Myc, Axin2, cyclin D1, and survivin Wnt target genes in the tumor tissues, as well as the induction of Ser33/Ser37/Thr41 phosphorylation of β-catenin, indicating that 4βHWE inhibits Wnt signaling in vivo to attenuate tumor proliferation.

Collectively, our results indicate that 4βHWE effectively suppressed the CRC growth in vitro and in vivo, at least in part, by blocking Wnt/β-catenin signaling, and that it can be of potential use in anti-tumor agent development as a novel Wnt signaling inhibitor.

## 4. Materials and Methods

### 4.1. Cell Culture 

All cell lines (HCT116, SW480, and HT-29), if not otherwise declared, were obtained from the Shanghai Institute of Biochemistry and Cell Biology, Chinese Academy of Sciences (Shanghai, China). The CCD-841-CoN normal human colonic epithelial cell line was a kind gift from Dr. Li (SIBS-CAS, Shanghai, China). Cells were maintained in medium according to the manufacturers’ instructions, supplemented with 10% (*v*/*v*) fetal bovine serum (HyClone), and penicillin/streptomycin (HyClone), in a humidified atmosphere with 5% CO_2_ at 37 °C. HEK-293W cells were cultured like the other mentioned cells but with the addition of 100 μg/mL G418 and 100 μg/mL Hygromycin B, as previously described [20].

### 4.2. Cell Transfection and Luciferase Reporter Assay

The dual-luciferase reporter assay was performed in two batches, one was in stably transfected HEK-293 cells stably transfected with SuperTopflash luciferase (ST-Luc), Wnt3a, and Renilla, and the other was with transient transfection using Lipofectamine™ 2000 (Invitrogen). Regarding the latter one, HCT116 and SW480 cells were seeded in a 96-well plate and grown to approximately 70% confluence, respectively. Next, 80 ng/well SuperTopflash (referred to as ST-Luc, kindly provided by Dr. Mao, KIZ-CAS, Kunming, China), a luciferase promoter construct containing TCF binding elements, was co-transfected with 8 ng/well Renilla control plasmid (pRL-SV40, Promega). After 3 h of transfection, the cells were exposed to a series of indicated concentrations of 4βHWE for another 24 h. Cells were then lysed and subjected to luciferase activity determination using the Dual-Luciferase Reporter Assay kit (Promega, Madison, WI, USA) following the manufacturer’s protocol.

### 4.3. Western Blotting Assay

For analysis of the whole cell lysate, SDS-PAGE buffer (62.5 mM Tris-HCl pH 6.8, 10% glycerol, 2% SDS, 50 mM DTT, and 0.01% bromophenol blue) was utilized to lyse cells after treatment. The subsequent SDS-PAGE and western blotting were carried out according to standard procedures [30]. Immunodetection was conducted using the following antibodies: anti-Survivin, anti-c-Myc, anti-Cyclin D1, anti-Axin 2, anti-PARP-1, anti-caspase 3, anti-cleaved caspase 3, anti-caspase 8 (Santa Cruz, CA, USA), anti-β-catenin (BD Biosciences), anti-phospho-β-catenin (Cell signaling Technology), anti-non-phospho (Active) β-catenin (Ser33/Ser37/Thr41) (Cell signaling Technology), anti-Lamin A/C (Epitomics), and anti-β-actin (Santa Cruz, CA, USA). Subsequently, the corresponding horseradish peroxidase (HRP)-conjugated secondary antibodies were used and protein bands were finally detected with LASmini 4000 (GE Healthcare, Barrington, IL, USA).

### 4.4. Nuclear and Cytoplasmic Fractionation

The collected cells were resuspended in ice-cold lysis buffer A (10 mM HEPES, 10 mM KCl, 1.5 mM MgCl2, 0.5 mM DTT, 0.4% NP-40, pH 7.9) with addition of an Ethylenediaminetetraacetic acid-free protease inhibitor cocktail (Roche). After 10 min of incubation on ice, the lysate was centrifuged for 5 min, at 800× *g*, 4 °C. The supernatant was collected as cytoplasmic fractionation. Pellets were washed 3 times with buffer A by 5 min of incubation, gentle pipetting, and spinning at 150× *g* for 2 min. The pelleted nuclei were resuspended in lysis buffer B (20 mM HEPES, pH 7.9, 420 mM NaCl, 0.5 mM DTT, 0.2 mM EDTA, and 25% glycerol) and incubated for 30 min on ice. Finally, the nuclear extraction was harvested after centrifugation at 12,000× *g*, 4 °C.

### 4.5. Small Interfering RNAs 

Duplex siRNAs were synthesized at GenePharma (Shanghai, China). The target sequences were as follows:

- Scrambled control: 5’-UUCUCCGAACGUGUCACGU-3’;
- *CTNNB1*: 5’-AGCUGAUAUUGAUGGACAG-3’.

### 4.6. Immunofluorescence Staining

The sample preparation has been described previously [31]. Briefly, drug-treated HCT 116 cells on coverslips were fixed in freshly-prepared 4% paraformaldehyde, permeabilized with 0.1% Triton X-100, and blocked with 3% BSA (Bovine Serum Albumin, BSA) in PBS (phosphate buffer saline, PBS). Afterwards, the cells were incubated with an anti-β-catenin antibody (BD Biosciences) overnight at 4 °C. Following 3 times washing with PBS, a FITC conjugated secondary antibody (Sigma-Aldrich) was employed for 1 h incubation at room temperature. Ten minutes prior to the next washing step, 4’,6-diamidino-2-phenylindole (DAPI) was added for nucleus staining. After washing and mounting procedures, the fluorescent images were examined under a microscope (Nikon, Tokyo, Japan). 

### 4.7. MTS Assay

The MTS assay was employed to assess the cytotoxicity of the studied compounds by using CellTiter 96^®^ AQueous One Solution Reagent (Promega). The manufacturer’s protocol was followed, which is described elsewhere [32]. Briefly, 5 × 10^3^ cells were seeded in a 96-well plate. After overnight settling, the indicated compounds and concentrations were added to the cells. After 48 h exposure, the CellTiter 96^®^ AQueous One Solution Reagent was directly added and incubated with the cells for 1–4 h, and then the optical density (OD) was recorded at 490 nm with a microplate reader (Bio-Rad Laboratories, Hercules, CA, USA).

### 4.8. Cell Cycle Analysis

HCT116 and HT-29 cells were exposed to indicated concentrations of 4βHWE for 24 h, respectively. Trypsin-digested cells were then collected and fixed with 70% ethanol overnight. After 3 times washing with pre-chilled PBS, the cells were stained with 50 μg/mL propidium iodide (PI) solution containing 50 μg/mL RNase, protected from light, and incubated at room temperature for 30 min. The samples were detected by FACSCalibur flow cytometry (BD Biosciences). The cell cycle distribution was processed by FlowJo V 7.6.1 software (Tree Star, San Carlos, CA, , USA, 2011). 

### 4.9. Cell Apoptosis Analysis

Cellular apoptosis was determined by the FITC Annexin V Apoptosis Detection Kit (BD Biosciences, San Jose, CA, USA) following the manufacturer’s instructions. After 48 h of treatment, both adherent and suspended cells were collected and washed with cold PBS. The cell pellet was resuspended in an appropriate volume of 1X binding buffer to achieve a recommended cell concentration of 1 × 10^6^ cells/ml. FITC Annexin V and PI were added, and then they were incubated at room temperature for 15 min while being protected from light. A calibration control was also set up. Fluorescent intensity was measured by FACSCalibur flow cytometry (BD Biosciences).

### 4.10. HCT116 Colorectal Xenografts

HCT116 cells (4 × 10^6^) were implanted subcutaneously into the right flank of the three-week-old BALB/c-nude mice (Vita River Laboratory Animal Technology Co.), according to published methods [32]. They were housed and treated according to the guidelines of the Institutional Animal Care and Use Committee. The mice were maintained under pathogen-free conditions. Tumor growth was monitored every other day by measuring two dimensions (L and W) with the following calculation formula: V (mm^3^) = (L × W^2^) × 0.5. When tumors reached a volume of ~90 mm^3^, the mice were randomized into two groups (*n* = 9/group) to receive treatment. One group was treated with normal saline as a control via intraperitoneal injection, while the other group underwent 5 mg/kg and 10 mg/kg of 4βHWE treatment. Tumor size and body weight were measured three times a week for a total of 14 days. The mice were euthanized, and the tumors were harvested 6 h after the final 4βHWE injection. The grafts were then weighed and stored at –80 °C for further western blotting analysis.
